# Poor agreement between teacher perceived and objective measures of weather conditions for school-based physical activity research

**DOI:** 10.1016/j.pmedr.2025.103328

**Published:** 2025-11-28

**Authors:** Yuzi Zhang, Kathryn G. Burford, Adriana Pérez, Kevin Lanza, Deborah Salvo, Brooklyn A. Baker, Deanna M. Hoelscher

**Affiliations:** a Michael & Susan Dell Center for Healthy Living, Austin, TX, United States of America; b The University of Texas Health Science Center at Houston (UTHealth Houston) School of Public Health, Austin, TX, United States of America; c Department of Kinesiology and Health Education, The University of Texas at Austin, Austin, TX, United States of America

**Keywords:** Safe routes to school, Weather, Climate change, Children, Active commuting to school

## Abstract

**Objective:**

To examine the level of agreement between teachers' perceptions of weather conditions within the National Safe Routes to School (SRTS) tally method with objective weather conditions.

**Methods:**

Secondary data analysis of the Safe Travel Environment Evaluation in Texas Schools (STREETS) study from January to April 2024 included 47 elementary schools in Central Texas. The third, fourth, and fifth-grade teachers reported perceived weather conditions (sunny, rainy, overcast, snow) in the morning and afternoon of each school day on the SRTS tally. National Oceanic and Atmospheric Administration Local Climatological data were used to calculate objective weather conditions (sunny, rainy, overcast) at the time closest to the start (7:10) and end (15:25) of school for the same days of SRTS tally measures. Kappa statistics and percent agreement assessed the level of agreement between perceived and objective weather conditions in the morning and afternoon for each tally day per grade.

**Results:**

For third-grade teachers, percent agreement between perceived and objective weather ranged from 0 to 14.3 % for sunny, 2.9–10.3 % for rainy, and 0–20 % for overcast. For fourth-grade teachers, the percent agreement ranged from 2.4 to 7.9 % for sunny, 2.6–12.2 % for rainy, and 0–13.2 % for overcast. For fifth-grade teachers, the percent agreement ranged from 2.5 to 8.1 % for sunny, 2.5–12.8 % for rainy, and 2.6–25 % for overcast. Kappa statistics ranged from −0.06 to 0.15, −0.10 to 0.07, and − 0.04 to 0.07 for grades three, four, and five.

**Conclusions:**

We found poor agreement between teachers' perceptions and objective measures of weather conditions.

## Highlights

• Teachers-reported weather showed poor agreement with objective weather data.
• Report timing and limited options may contribute to variability in reported weather.
• Combining objective and teacher-reported weather data is recommended.

## 1 Introduction

According to the 2018 Physical Activity Guidelines for Americans, children and adolescents should engage in 60 min or more of daily moderate-to-vigorous physical activity. Youth who engage in higher intensity and amounts of physical activity experience improved cardiometabolic health, bone health, cardiorespiratory fitness, and adiposity status, among other health benefits (Janssen and LeBlanc, 2010). However, only 20–28 % of United States (U.S.) children and youth ages 6–17 met the Physical Activity Guidelines between 2021 and 2022 (National Physical Activity Plan Alliance, 2018). In Texas, less than half (43 %) of the children accumulate 60 min of daily moderate-to-vigorous physical activity at least five days per week (National Physical Activity Plan Alliance, 2018). Of particular concern is that the prevalence of children and youth meeting guidelines has been steadily declining overtime, suggesting that little, if any, progress has been made to increase population-level physical inactivity among children and adolescents. Thus, there remains an urgent need to identify scalable policies and programs that address this growing public health crisis.

Schools are one of the primary settings where children and youth can participate in physical activity, such as during physical education class, organized or unorganized team or individual sports, active play, and active commuting to school (ACS) (National Physical Activity Plan Alliance, 2018). ACS, or walking, bicycling, or rolling to and from school, is an important opportunity for children to participate in physical activity because it can be integrated into children and adolescent's daily routines (National Physical Activity Plan Alliance, 2018). In fact, a systematic review indicated that during the home-to-school and school-to-home trips, approximately nine and 20 min of moderate-to-vigorous physical activity could be accumulated, respectively, which contributed to about 15 % and 33 % of the daily physical activity recommendations for children (Campos-Garzón et al., 2023). Despite the potential contribution of ACS to children's daily physical activity, only 11 % of U.S. children aged 5–17 walked or biked to school in 2017, representing a significant decline from 41 % in 1969 (Kontou et al., 2020; McDonald, 2007).

Children's ACS is influenced by individual, household, built, natural, social environment, and policy factors that are often the targets of school-based active transportation interventions such as Safe Routes to School (SRTS) programs (Burford et al., 2025; Mitra, 2013; Panter et al., 2008). However, an understudied but key barrier to ACS, as well as other outdoor physical activity behaviors, is the weather (Zheng et al., 2021). As more frequent and intense extreme weather and changes to the weather conditions are expected to continue to occur as a consequence of climate change, it is critical that we understand the impact of weather-related barriers on children's school-based physical activity behaviors. These data can be used to educate school communities and help develop school-based climate adaptation strategies (Hyndman and Vanos, 2023; Reidmiller et al., 2019). Importantly, a stronger evidence base would help develop school policies related to extreme-weather protection.

Most studies linking various weather conditions, such as precipitation, temperature, hours of sunlight, and windspeed, with ACS found no association (Blanchette et al., 2021; Chillón et al., 2014; Helbich et al., 2016; Herrador-Colmenero et al., 2018; Mitra and Faulkner, 2012; Oliver et al., 2014; Robertson-Wilson et al., 2008; Sirard et al., 2005). A major limitation across the existing studies is that weather measurements are averaged over a given day, week, or month, rather than time-matching weather to when children ACS. We found only one study to address this measurement limitation, by matching data to the closest weather station with the time children actually walk and bike to school. However, this time-matched approach requires advanced data skills and resources to implement (Helbich et al., 2016). Thus, there remains a need for measure that school-communities can use to report on weather-related barriers for ACS (Omura et al., 2019).

The National SRTS tally is a valid and reliable method capturing school-level walking and bicycling to and from school, which includes a measure asking teachers to report the weather conditions in the morning and afternoon of a school day (McDonald et al., 2011). As the SRTS tally method was developed for evaluation efforts of SRTS programs and projects across the U.S. (Hoelscher et al., 2022), this measure has the potential to be an efficient, practical, and scalable method for capturing weather-related barriers to children's ACS during different climates. However, there are no studies to evaluate the agreement of teacher's perception of the weather conditions with an objective measure of weather conditions during the time children walk or bike to and from school to determine the feasibility of using the SRTS tally method for measuring weather conditions. Thus, the purpose of this study was to determine the level of agreement between teachers' perceptions of the weather conditions within the National SRTS tally and objective weather conditions using National Oceanic and Atmospheric Administration (NOAA) Local Climatological data in a U.S. city with a warm climate.

## 2 Methods

### 2.1 Data source and study design

Secondary data analyses of the Safe Travel Environment Evaluation in Texas Schools (STREETS) serial cross-sectional study were conducted, which is a five-year natural experiment (2018–2023) to evaluate the impact of SRTS infrastructure projects funded by a 2016 bond initiative from the City of Austin on population-level changes in the prevalence of ACS in elementary schools in Central Texas. The complete methods of the STREETS study have been presented elsewhere (Hoelscher et al., 2022). The current study used the latest SRTS Tally data, which was collected from January to April 2024, from 47 elementary schools in Central Texas. Informed consent procedures for principals and classroom teachers and study protocol were approved by the institutional review board at The University of Texas Health Science Center at Houston (HSC-SPH-17-0638) and by the research departments at each participating school district.

### 2.2 Measures

#### 2.2.1 Teachers' perceptions of weather conditions

Teacher-perceived weather data were collected using the standard SRTS tally method developed by the National Center for SRTS (SRTS Guide, 2025). Teachers in grades three to five classrooms reported the weather conditions during the morning and afternoon of each SRTS tally day. Tally days were designated as Tuesday, Wednesday, and Thursday, with the intent that each school would collect three days of data. Weather conditions included multiple-choice options: sunny, rainy, overcast, and snow. We computed a school-level weather condition variable for the analysis by identifying the mode weather condition per school (*N* = 65), grade (third-fifth grade), date, and time (am, pm). The school-level subjective weather condition (three categories: sunny, rainy, and overcast) was the mode weather condition based on the classroom-level observations. The school-level subjective weather at this school, grade, date, and time would be excluded from the analysis if no mode could be determined due to the lack of consensus among teachers' perception of weather within the same school and grade for the specific date and time.

#### 2.2.2 Objective weather conditions

Objective weather data were collected from the NOAA Local Climatological Data (Local Climatological Data | Data Tools | Climate Data Online | National Climatic Data Center, 2025). Each school was matched to the weather station with the closest distance using a Geographic Information System (ArcGIS 10.8, ESRI, Redlands, CA, USA). For the closest weather station to each school, we obtained two hourly weather variables from NOAA, precipitation and sky condition, corresponding to the tally data collection dates between January and April 2024. Elementary schools in our sample started between 7:15 and 7:40, and end between 14:20 and 15:10. However, most elementary schools in our study sample started their school day at 7:40 (72 % of schools) and ended their school day at 15:10 (68 % of schools). Thus, we selected 7:10 and 15:25 as the active commuting to school start and end times or 30 min from the school start time and 15 min from the school end time. We chose these time periods to approximate the time parents make decisions about school transportation modes, which is a complex process that is largely dependent on parental perceptions of factors influencing time on any given day (e.g., work schedule) (Faulkner et al., 2010). Additionally, the vast majority of U.S. children walk and bike less than 1 mile to school (about a 20-min walk and 10-min bike) (Kontou et al., 2020). The hourly precipitation and the hourly sky conditions at the time closest to the decision time in the morning (7:10) and the afternoon (15:25) were obtained, corresponding to the same days of the SRTS tally measures, reflecting precipitation and sky conditions for the morning and the afternoon.

If any trace amounts of precipitation were reported, a new binary precipitation variable was classified as rainy versus not rainy (Supplement Table 1). If the sky condition was less than or equal to 50 % of the sky covered by clouds, then a new binary variable, sky condition, was classified as sunny. If the sky condition was over 50 % of the sky covered by clouds or obscured sky, the binary sky condition was classified as overcast (Local Climatological Data Dataset Documentation, 2025; NOAA, 2025). We then created a new categorical weather variable with three levels (rainy, sunny, and overcast) by combining the binary precipitation and the binary sky condition in the morning and afternoon, respectively. The new categorical weather variable was defined as rainy if the binary precipitation variable was ever defined as rainy; if the binary precipitation variable was not rainy and the binary sky condition variable was sunny, the new categorical weather variable was defined as sunny; if the binary precipitation variable was not rainy and the binary sky condition variable was defined as overcast, then the new categorical weather variable was defined as overcast.

#### 2.2.3 Other variables

School-level characteristics were obtained from the Texas Education Agency for academic year 2023–2024. These school-level characteristics included total school enrollment, proportion of girls, racial and ethnic distribution of students determined by the school district, the percentage of economically disadvantaged students (eligible for free or reduced lunch), percentage of students with limited English proficiency, and community type (major urban, urban).

#### 2.2.4 Data analysis

Descriptive statistics were used to calculate means and standard deviations (SD), for continuous school-level characteristics, and frequencies with proportions for categorical characteristics. Kappa test statistics and percent agreement were calculated to assess the level of agreement between perceived and objective weather conditions by morning and afternoon for each tally day per each grade (McHugh, 2012). Kappa statistics were interpreted as: almost perfect (>0.90), strong (0.80–0.90), moderate (0.60–0.79), weak (0.40–0.59), minimal (0.21–0.39), and none (0–0.20) (McHugh, 2012). All data analyses were performed using R (version 4.3.0) and SAS (OnDemand for Academics).

As the current study is part of a natural experiment, teachers' perceptions and children's active commuting behaviors may be affected by the SRTS infrastructure change and the COVID-19 pandemic. A sensitivity analysis was conducted using the baseline SRTS Tally data collected between January and May 2019, before the infrastructure improvement and the pandemic.

## 3 Results

Of the 47 schools, the average number of students enrolled per school in the academic year of 2018–2019 was 505.4 (SD = 197.2), with most students being Hispanic (53.8 %) and economically disadvantaged (53.3 %) (Table 1).

**Table 1:** School-level Characteristics from the Texas Education Agency data, 2023–2024 school year (*N* = 47).

Characteristics	Mean (SD)/n (%)
Total school enrollment	505.4 (197.2)
Percent girl	48.4 (2.8)
Percent racial/ethnic distribution	
African American	6.7 (7.2)
Hispanic	53.8 (26.3)
White or other, non-Hispanic	39.4 (27.6)
Percent economically disadvantage students	53.3 (31.6)
Percent students with limited English proficiency	41.8 (25.8)
Community type	
Major urban	38 (80.9 %)
Urban	9 (19.1 %)

Note. SD = Standard Deviation.

**Fig. 1:**
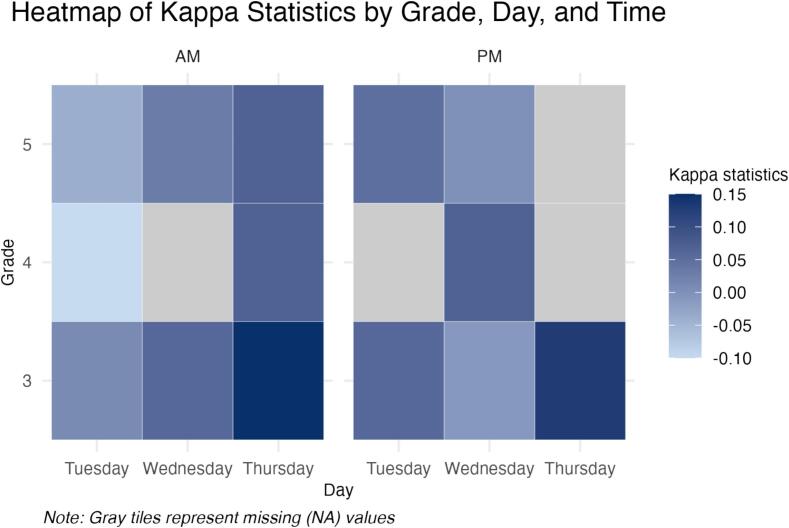
Heatmap of Kappa Statistics (by grade, day, and time) of the subjective teacher-perceived and objective weather condition metrics from schools in Central Texas, 2024.

For each grade at a specific weekday and time, the number of schools that had a consensus of teachers' perceived weather conditions ranged from 34 schools (third grade on Tuesday AM) to 41 schools (fourth grade on Wednesday AM) (Table 2). Overall, the percent agreement between the teacher-perceived weather condition measure and the objective measure ranging from 0 % to 25 %. The highest level of agreement was measured for overcast (range: 0 %–25 %) days, while the agreement tended to be lower for sunny (range: 0 %–14.3 %) and rainy (2.5 %–12.8 %) days. These percentages did not vary appreciably by grade level. Kappa statistics revealed no agreement between the teacher-perceived and objective metrics, with Kappa scores ranging from −0.06 to 0.15, −0.10 to 0.07, and − 0.04 to 0.07 for third, fourth, and fifth grades, respectively (Fig. 1). Because no sunny conditions were observed for schools for grade four on Thursday in the PM, and no rainy conditions were observed for schools for grade four on Tuesday in the PM, and grade five on Thursday PM, the percent agreement of sunny and rainy conditions and the Kappa statistics could not be calculated and showed as non-applicable.

**Table 2:** Percent Agreement and Kappa statistics (per grade, per day, and time) of the subjective teacher-perceived and objective weather condition metrics from schools in Central Texas, 2024.

Grade	Day	Time	Number of schools	%Agreement of Sunny	%Agreement of Rainy	%Agreement of Overcast	Kappa Statistic (95 % CI)
3	Tuesday	AM	34	0.0	8.8	8.8	0.01 (−0.11, 0.12)
		PM	40	5.0	5.0	5.0	0.06 (0.00, 0.13)
	Wednesday	AM	39	0.0	10.3	0.0	−0.06 (−0.14, 0.02)
		PM	37	2.7	5.4	5.4	−0.01 (−0.12, 0.11)
	Thursday	AM	35	14.3	2.9	8.6	0.15 (0.05, 0.25)
		PM	35	0.0	5.7	20.0	0.13 (0.02, 0.24)
4	Tuesday	AM	35	2.9	2.9	2.9	−0.10 (−0.26, 0.05)
		PM	38	5.3	NA	5.3	NA
	Wednesday	AM	41	2.4	12.2	0.0	−0.03 (−0.12, 0.05)
		PM	39	2.6	7.7	10.3	0.07 (−0.04, 0.17)
	Thursday	AM	38	7.9	2.6	7.9	0.07 (−0.02, 0.16)
		PM	38	NA	5.3	13.2	NA
5	Tuesday	AM	39	2.6	5.1	2.6	−0.04 (−0.15, 0.06)
		PM	40	5.0	2.5	5.0	0.05 (−0.01, 0.12)
	Wednesday	AM	39	2.6	12.8	2.6	0.03 (−0.03, 0.09)
		PM	36	2.8	2.8	8.3	0.00 (−0.12, 0.13)
	Thursday	AM	37	8.1	5.4	5.4	0.07 (−0.02, 0.16)
		PM	40?	2.5	NA	25.0	NA

*Note.* CI = Confidence Interval, NA = non-Applicable.

The sensitivity analysis included 65 elementary schools, with most students being Hispanic and economically disadvantaged (Supplement Table 2). Kappa statistics revealed minimal to moderate agreement between teacher-perceived and objective metrics, with Kappa scores ranging from 0.38 to 0.60 (Supplement Table 3, Supplement Fig. 1).

## 4 Discussion

This study examined the level of agreement between elementary school teacher-perceived weather and objectively measured weather conditions from weather stations closest schools. In sum, we found almost no agreement between teacher-perceived weather and objectively measured weather conditions. In the sensitivity analysis, the agreements were higher with the baseline data; however, the Kappa statistics were still considered minimal to moderate. Therefore, this poor agreement suggests that if subjective weather is solely used as an exposure, effect estimates of weather on ACS would likely be biased.

While the SRTS Tally method is a feasible approach for practitioners and school communities to use in evaluation efforts, especially when compared to the objective method we implemented in our study, there are several methodological limitations that limit its utility for active living research. First, the measure does not ask teachers to provide the exact time to report the weather. Thus, the perceived weather condition metric does not necessarily represent the weather conditions at the time a student walked or biked to school, but rather the time the teacher completed the form. Second, data from multiple teachers for each grade were collected and teachers likely reported weather conditions for different times of the day, which would lead to substantial variability in reported weather conditions. Asking all teachers to report the weather conditions may be cumbersome, given teachers' existing workload and especially for schools with limited resources. Additionally, only four weather conditions were included on the tally form - sunny, rainy, overcast, and snowy – which likely lowers the burden on teachers, but these categories are inherently subjective. These existing weather conditions also do not capture other potentially more pertinent weather-related barriers, such as extreme heat or heavy precipitation. Lastly, analyzing these data required advanced data science skills, and results may be difficult to translate to stakeholders. In this study, we derived the mode weather condition for each grade, by specific day and time, to represent the school-level weather condition. However, the mode weather condition could not be obtained if there was no consensus among the classrooms, resulting in 5 % to 22 % of teachers being excluded across grade, day, and time in 2024 (Supplement Table 4; Supplement Table 5 for 2019 data).

To address the described limitations of the national SRTS Tally method and improve the reporting fidelity, we have developed a set of recommendations. First, we suggest that only one teacher per grade report the weather conditions, which would limit the burden to schools, especially those with limited resources, and reduce variability in reporting overall. Second, an additional real-time objective measure could be used by the teachers, such as weather data captured from smartphone applications. Third, teachers, school stakeholders, parents, and children could also be engaged in future research to develop an item(s) that measures the most relevant weather-related barriers to active travel, such as extreme weather events. Lastly, teachers should be provided with training on the tool prior to data collection and with clear instructions that define the weather conditions of interest (e.g., what constitutes “overcast”).

We found no previous studies have examined the agreement between weather measured with the SRTS tally and objective weather measurements. Nevertheless, the “mismatch” between perceived environment attributes and objective measures of the environment is a common finding. McGinn et al. (2007) found that there was no agreement (Kappa statistic = 0.00, 95 %CI = -0.02, 0.03) between the perception of weather as a problem in the neighborhood and the number of daily adverse weather events, including precipitation, negative standard pressure, wind chill, or heat index, determined using local climatological data (McGinn et al., 2007). Furthermore, there was no agreement between the perception of weather as a barrier to physical activity and the objective weather measures (Kappa statistic = 0.03, 95 %CI = -0.02, 0.07) (McGinn et al., 2007). Additionally, the agreement between perceived and objective weather was not modified by individuals' PA levels, suggesting that a better understanding of what factors influence the perception and contribute to the divergence between subjective and objective measured weather could shed light on developing future constructs for large-scale studies (McGinn et al., 2007). Weather is not the only case; findings from a systematic review indicated that among the studies examined, 72 % of the pairs of perceived and objective neighborhood environment variables, such as public transit and safety from crime, showed poor (kappa statistic<0.0) or slight (kappa statistic = 0.0–0.2) agreement (McGinn et al., 2007; Orstad et al., 2017). Some environmental attributes exhibited high variability on the percent agreement and Kappa statistics; for example, the Kappa statistics ranged from −0.07 to 0.66 for recreation facilities, indicating that characteristics of the study population and neighborhood context could affect the agreement between the subjective and objective measures (Orstad et al., 2017). As a whole, findings from this work, along with ours, seem to suggest that the subjective and objective measures of the same built and natural environment construct (i.e., weather conditions) are actually unique, and have distinct causal pathways (e.g., perceived risk, classroom routes). This justifies the joint measurement of objective and subjective measures of weather rather than substitution within analytic studies on determinants of ACS and evaluations of SRTS programs. (Aranda-Balboa et al., 2021; Carlson et al., 2021; Chillón et al., 2014; Durand et al., 2017; Klos et al., 2023; Lanza et al., 2020; Lanza and Durand, 2021; Panter et al., 2008).

Several limitations associated with the objective measure of weather conditions should also be noted. Although we matched the weather data based on the location and the school start and end time, there remains variability between the distance for each school and the closest weather station. Therefore, the objective measure of weather data may not represent the exact weather conditions of a school neighborhood. Furthermore, we considered using 30 min before school starts and 15 min after school ends to approximate the time parents make decisions about school transportation modes, and that the majority of children are walking or bicycling to school within a 10–20 min time period (Kontou et al., 2020). However, parental decisions might occur at a different time, such as the night before, and rely on a weather forecast or other unique barriers that impact school travel timing. Thus, future work could explore if results are dependent on school travel window choices, although it is unlikely NOAA data change substantially within ±15 min of our existing windows. The specific time the teachers conducted the tally and reported the weather conditions was not recorded, which as previously discussed, is a limitation of this measure. Therefore, there could be a mismatch between the time teachers conducted the tally and the time we used to obtain data from weather stations. Lastly, our sensitivity analysis using baseline data indicated slightly improved agreement statistics, which is likely explained by the increased sample of schools at baseline (65 vs. 47 schools) and longitudinal study fatigue by teachers throughout the STREETS project. As even more agreement statistics could not be calculated for 2024 compared to baseline due to the lack of data (Fig. 1 and Supplemental Fig. 1), this only further suggests that a more feasible method for capturing reported weather is needed.

## 5 Conclusions

This study found poor agreement between teacher-perceived weather conditions from the SRTS Tally and objectively measured weather from the closest weather station. While the national SRTS tally method has potential efficiency and practicality gains for measuring weather conditions, the poor agreement suggests that it is not an appropriate proxy for the objective measure. Ideally, valid measures of both objective and perceived weather constructs are included as exposures in studies of ACS. We suggest opportunities to improve the feasibility of this method, including using weather APIs, centralized reporting, teacher training, and working with the school community to develop more salient items to measure extreme weather conditions. As the SRTS tally method is an important evaluation metric for SRTS programs, improving the reporting fidelity of this method is essential moving forward, or alternatively, developing an easier way to collect weather condition data during the school commute times. These next research steps are especially pertinent given the increasing threats of adverse or changing weather events and their potential impact on children's physical activity behaviors.

## CRediT authorship contribution statement

**Yuzi Zhang:** Writing – review & editing, Writing – original draft, Methodology, Investigation, Formal analysis, Data curation, Conceptualization. **Kathryn G. Burford:** Writing – review & editing, Writing – original draft, Methodology, Investigation, Formal analysis, Data curation, Conceptualization. **Adriana Pérez:** Writing – review & editing, Methodology, Investigation, Formal analysis, Data curation, Conceptualization. **Kevin Lanza:** Writing – review & editing, Methodology, Investigation, Conceptualization. **Deborah Salvo:** Writing – review & editing, Methodology, Conceptualization. **Brooklyn A. Baker:** Writing – review & editing. **Deanna M. Hoelscher:** Writing – review & editing, Supervision, Funding acquisition.

## Informed consent statement

Informed consent was obtained from all schools and individuals involved in the study.

## Institutional review board statement

This study was approved by the Institutional Review Board (or Ethics Committee) of UTHealth (HSC-SPH-17-0638 and 11 August 2017).

## Funding

This research was funded by the Eunice Kennedy Shriver National Institute of Child Health & Human Development, grant number R01 HD097669, and support was provided by the Michael and Susan Dell Foundation through the Michael & Susan Dell Center for Healthy Living. Dr. Lanza is supported by the National Institute of Environmental Health Sciences of the National Institutes of Health under award number K01ES034382.

## Declaration of competing interest

The authors declare that they have no known competing financial interests or personal relationships that could have appeared to influence the work reported in this paper.

## Data Availability

Data will be made available on request.

## Appendix A Supplementary data

Supplementary material Image 1
