# Supplementary material for: Poor agreement between teacher perceived and objective measures of weather conditions for school-based physical activity research
Source: Prev Med Rep. 2025 Nov 28;61:103328. doi: 10.1016/j.pmedr.2025.103328 (PMC12719780; doi:10.1016/j.pmedr.2025.103328)

| Supplement Table 1. Decision rules for creating the objective weather variable based on the National Oceanic and Atmospheric Administration Local Climatological data. | | |
| --- | --- | --- |
| **Variable** | **Note** | **Decision Rules** |
| Hourly sky conditions | Original NOAA variables | NA |
| Hourly precipitation | Original NOAA variables | NA |
| Binary sky conditions (sunny, overcast) | Binary variable, computed by Hourly sky condition | 1. If hourly sky conditions coded as CLR, FEW, SCT, then binary sky conditions coded as “sunny”; 2. If hourly sky condition coded as BKN, OVC, VV, or 10, then binary sky conditions coded as “overcast”; 3. If hourly sky conditions were null (blank) or number only, then these values were missing. |
| Binary precipitation (rainy, not rainy) | Binary variable, computed by hourly precipitation | 1. If hourly precipitation coded as T or with number, then coded as “rainy”; 2. If hourly precipitation were null (blank), then coded as “not rainy”; 3. If hourly precipitation coded as M, then these values were missing. |
| Objective weather variable (rainy, sunny, overcast) | 3-level categorical variable, computed by binary sky conditions and binary precipitation | 1. If binary precipitation coded as rainy, then coded as “rainy”; 2. If binary precipitation coded as not rainy and binary sky conditions coded as sunny, then coded as “sunny”; 3. If binary precipitation as not rainy and binary sky conditions coded as overcast, then coded as “overcast”. |
| *Note.*  Abbreviation: NOAA = National Oceanic and Atmospheric Administration, NA = Not Applicable.  Code names from NOAA Local Climatological data documentation: CLR = clear sky, FEW = few clouds, SCT = scattered clouds, BKN = broken clouds, OVC = overcast, VV = obscured sky, 10 = partially obscured sky, T = Trace amounts of precipitation, M = missing. | | |

| Supplement Table 2. School-level Characteristics from the 2018-2019 Texas Education Agency data (N=65) | |
| --- | --- |
| Characteristics | Mean (SD)/n (%) |
| Total school enrollment | 557.1 (192.0) |
| Percent girls | 48.6 (2.6) |
| Percent racial/ethnic distribution |  |
| African American | 4.3 (5.1) |
| Hispanic | 64.0 (32.4) |
| White or other, non-Hispanic | 31.0 (33.2) |
| Percent economically disadvantaged students | 55.6 (31.1) |
| Percent students with limited English proficiency | 34.8 (26.9) |
| Community type | N (%) |
| Major urban | 51 (78.5%) |
| Urban | 14 (21.5%) |
| *Note. SD = Standard Deviation* | |

| Supplement Table 3. Percent Agreement and Kappa statistics (per grade, per day, and time) of the subjective teacher-perceived and objective weather condition metrics from schools in Central Texas, 2019 | | | | | | | |
| --- | --- | --- | --- | --- | --- | --- | --- |
| Grade | Day | Time | Number of schools | %Agreement of Sunny | %Agreement of Rainy | %Agreement of Overcast | Kappa Statistic (95% CI) |
| 3 | Tuesday | AM | 56 | 16.1 | 5.4 | 48.2 | 0.42 (0.19, 0.64) |
|  |  | PM | 54 | 31.5 | 0 | 44.4 | 0.53 (0.32, 0.74) |
|  | Wednesday | AM | 53 | 17.0 | 28.3 | 28.3 | 0.60 (0.42, 0.78) |
|  |  | PM | 57 | 28.1 | 17.5 | 15.8 | 0.41 (0.22, 0.60) |
|  | Thursday | AM | 56 | 21.4 | 1.8 | 46.4 | 0.43 (0.22, 0.63) |
|  |  | PM | 56 | 62.5 | 1.8 | 10.7 | 0.40 (0.17, 0.64) |
| 4 | Tuesday | AM | 59 | 13.6 | 6.8 | 47.5 | 0.40 (0.18, 0.62) |
|  |  | PM | 52 | 26.9 | 0 | 40.4 | 0.39 (0.18, 0.61) |
|  | Wednesday | AM | 60 | 11.7 | 25.0 | 31.7 | 0.50 (0.31, 0.69) |
|  |  | PM | 56 | 26.8 | 21.4 | 17.9 | 0.49 (0.30, 0.68) |
|  | Thursday | AM | 58 | 31.0 | 0 | 32.8 | 0.38 (0.19, 0.56) |
|  |  | PM | 59 | 66.1 | 1.7 | 11.9 | 0.51 (0.29, 0.72) |
| 5 | Tuesday | AM | 50 | 16.0 | 4.0 | 56.0 | 0.51 (0.28, 0.74) |
|  |  | PM | 49 | 22.5 | NA | 49.0 | NA |
|  | Wednesday | AM | 52 | 15.4 | 25.0 | 30.8 | 0.55 (0.36, 0.75) |
|  |  | PM | 50 | 22.0 | 22.0 | 16.0 | 0.40 (0.20, 0.60) |
|  | Thursday | AM | 48 | 31.3 | 0 | 39.6 | 0.48 (0.28, 0.69) |
|  |  | PM | 50 | 52.0 | 2.0 | 22.0 | 0.53 (0.31, 0.75) |
| Note. CI = Confidence Interval, NA = Not-Applicable | | | | | | | |

| Supplement Table 4. The number of schools and teachers in the analysis and the number and proportion excluded due to lack of consensus, from schools in Central Texas, 2024 | | | | | | | | | | |
| --- | --- | --- | --- | --- | --- | --- | --- | --- | --- | --- |
| Grade | Day | Time | Schools submitted tally data (n) | Teachers in classroom submitted tally data (n) | Schools in the analysis (n) | Teachers in classrooms in the analysis (n) | Schools excluded (n)^1^ | Teachers in classroom excluded (n)^1^ | Proportion of schools excluded^1^ | Proportion of teachers in classroom excluded^1^ |
| 3 | Tue | AM | 44 | 139 | 34 | 112 | 10 | 27 | 23% | 19% |
|  |  | PM | 44 | 139 | 40 | 129 | 4 | 10 | 9% | 7% |
|  | Wed | AM | 44 | 139 | 39 | 124 | 5 | 15 | 11% | 11% |
|  |  | PM | 44 | 139 | 37 | 120 | 7 | 19 | 16% | 14% |
|  | Thur | AM | 44 | 139 | 35 | 108 | 9 | 31 | 20% | 22% |
|  |  | PM | 44 | 139 | 35 | 113 | 9 | 26 | 20% | 19% |
| 4 | Tue | AM | 44 | 133 | 35 | 105 | 9 | 28 | 20% | 21% |
|  |  | PM | 44 | 133 | 38 | 121 | 6 | 12 | 14% | 9% |
|  | Wed | AM | 44 | 133 | 41 | 126 | 3 | 7 | 7% | 5% |
|  |  | PM | 44 | 133 | 39 | 122 | 5 | 11 | 11% | 8% |
|  | Thur | AM | 44 | 133 | 38 | 111 | 6 | 22 | 14% | 17% |
|  |  | PM | 44 | 133 | 38 | 119 | 6 | 14 | 14% | 11% |
| 5 | Tue | AM | 45 | 123 | 39 | 107 | 6 | 16 | 13% | 13% |
|  |  | PM | 45 | 123 | 40 | 111 | 5 | 12 | 11% | 10% |
|  | Wed | AM | 45 | 123 | 39 | 105 | 6 | 18 | 13% | 15% |
|  |  | PM | 45 | 123 | 36 | 98 | 9 | 25 | 20% | 20% |
|  | Thur | AM | 45 | 123 | 37 | 100 | 8 | 23 | 18% | 19% |
|  |  | PM | 45 | 123 | 40 | 108 | 5 | 15 | 11% | 12% |
| *Note.* ^1^The exclusion due to lack of consensus among teachers' perception of weather within the same school and grade for the specific date and time. Tue = Tuesday, Wed = Wednesday, Thur = Thursday | | | | | | | | | | |

| Supplement Table 5. The number of schools and teachers in the analysis and the number and proportion excluded due to lack of consensus, from schools in Central Texas, 2019 | | | | | | | | | | |
| --- | --- | --- | --- | --- | --- | --- | --- | --- | --- | --- |
| Grade | Day | Time | Schools submitted tally data (n) | Teachers in classroom submitted tally data (n) | Schools in the analysis (n) | Teachers in classrooms in the analysis (n) | Schools excluded (n)^1^ | Teachers in classroom excluded (n)^1^ | Proportion of schools excluded^1^ | Proportion of teachers in classroom excluded^1^ |
| 3 | Tue | AM | 65 | 256 | 56 | 216 | 9 | 40 | 14% | 16% |
|  |  | PM | 65 | 256 | 54 | 219 | 11 | 37 | 17% | 14% |
|  | Wed | AM | 65 | 256 | 53 | 202 | 12 | 54 | 18% | 21% |
|  |  | PM | 65 | 256 | 57 | 218 | 8 | 38 | 12% | 15% |
|  | Thur | AM | 65 | 256 | 56 | 219 | 9 | 37 | 14% | 14% |
|  |  | PM | 65 | 256 | 56 | 220 | 9 | 36 | 14% | 14% |
| 4 | Tue | AM | 65 | 259 | 59 | 238 | 6 | 21 | 9% | 8% |
|  |  | PM | 65 | 259 | 52 | 211 | 13 | 48 | 20% | 19% |
|  | Wed | AM | 65 | 259 | 60 | 239 | 5 | 20 | 8% | 8% |
|  |  | PM | 65 | 259 | 56 | 227 | 9 | 32 | 14% | 12% |
|  | Thur | AM | 65 | 259 | 58 | 235 | 7 | 24 | 11% | 9% |
|  |  | PM | 65 | 259 | 59 | 237 | 6 | 22 | 9% | 8% |
| 5 | Tue | AM | 58 | 179 | 50 | 157 | 8 | 22 | 14% | 12% |
|  |  | PM | 58 | 179 | 49 | 157 | 9 | 22 | 16% | 12% |
|  | Wed | AM | 58 | 179 | 52 | 154 | 6 | 25 | 10% | 14% |
|  |  | PM | 58 | 179 | 50 | 151 | 8 | 28 | 14% | 16% |
|  | Thur | AM | 58 | 179 | 48 | 147 | 10 | 32 | 17% | 18% |
|  |  | PM | 58 | 179 | 50 | 159 | 8 | 20 | 14% | 11% |
| Note. ^1^The exclusion due to lack of consensus among teachers' perception of weather within the same school and grade for the specific date and time. Tue = Tuesday, Wed = Wednesday, Thur = Thursday | | | | | | | | | | |

Supplement Figure 1. Heatmap of Kappa Statistics (by grade, day, and time) of the subjective teacher-perceived and objective weather condition metrics from schools in Central Texas, 2019


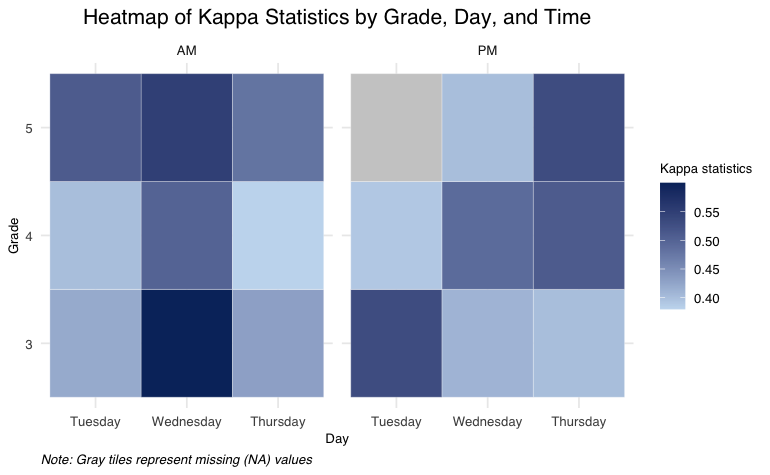

Supplement: Supplementary file 1 — Supplementary material [file mmc1.docx]
